# Higher Leptin but Not Human Milk Macronutrient Concentration Distinguishes Normal-Weight from Obese Mothers at 1-Month Postpartum

**DOI:** 10.1371/journal.pone.0168568

**Published:** 2016-12-22

**Authors:** Arnaud De Luca, Marine Frasquet-Darrieux, Marie-Agnès Gaud, Patricia Christin, Clair-Yves Boquien, Christine Millet, Manon Herviou, Dominique Darmaun, Richard J. Robins, Pierre Ingrand, Régis Hankard

**Affiliations:** 1 INSERM U 1069, Tours, France; 2 University Hospital of Tours, Tours, France; 3 INSERM CIC 1402, Poitiers, France; 4 University of Poitiers, Poitiers, France; 5 Pediatrics-Child Nutrition, University Hospital of Poitiers, Poitiers, France; 6 Maternity, General Hospital of Chatellerault, Chatellerault, France; 7 INRA UMR 1280, Institut des Maladies de l’Appareil Digestif, University Hospital of Nantes, Nantes, France; 8 Centre de Recherche en Nutrition Humaine Ouest, Nantes, France; 9 Nuclear Medicine Laboratory, University Hospital of Poitiers, Poitiers, France; 10 Elucidation of Biosynthesis by Isotopic Spectrometry Group, CEISAM, CNRS-University of Nantes UMR 6230, Nantes, France; 11 University F Rabelais, Tours, France; University Medical Center Utrecht, NETHERLANDS

## Abstract

**Introduction:**

Exclusively breastfed infants born to obese mothers have previously been shown to gain less weight by 1-month postpartum than infants of normal-weight mothers. Our hypothesis is that human milk composition and volume may differ between obese and normal-weight mothers.

**Objective:**

To compare human milk leptin, macronutrient concentration, and volume in obese and normal-weight mothers. Mother and infant characteristics were studied as secondary aims.

**Materials and Methods:**

This cross-sectional observational study compared 50 obese mothers matched for age, parity, ethnic origin, and educational level with 50 normal-weight mothers. Leptin, macronutrient human milk concentration, and milk volume were determined at 1 month in exclusively breastfed infants. Mother characteristics and infant growth were recorded.

**Results:**

Human milk leptin concentration was higher in obese mothers than normal-weight mothers (4.8±2.7 vs. 2.5±1.5 ng.mL^-1^, p<0.001). No difference was observed between obese and normal-weight mothers in protein, lipid, carbohydrate content, and volume, nor in infant weight gain.

**Conclusion:**

Leptin concentration was higher in the milk of obese mothers than that of normal-weight mothers, but macronutrient concentration was not. It remains to be established whether the higher leptin content impacts on infant growth beyond the 1-month of the study period.

## Introduction

Obesity affects the outcome of pregnancy on both the maternal and fetal sides and contributes to the health-status of the offspring at adulthood [1]. A growing number of women of childbearing age are obese or overweight [2]. Obese women or women with excessive gestational weight gain are less prone to initiate breastfeeding than normal-weight women [3,4]. They are also more at risk of having breastfeeding difficulties, which may lead to discontinuing breastfeeding [5–8]. Physiological factors such as delayed lactogenesis or lower prolactin response to sucking have been identified, but psycho-social factors are also involved [5,9,10]. Within the Inserm EDEN cohort study of the pre- and early-postnatal determinants of child health and development, we have observed that exclusively breastfed infants born to obese mothers showed a lower infant weight gain (IWG) during the first month of postnatal life [8,11]. The effect was small and transient. A number of nutritional factors could contribute to differential weight gain, notably the fat/carbohydrate content and the related energy availability, and/or the appetite-suppressing effect of leptin (causing babies to get satisfied faster). Although a positive correlation between human milk leptin concentration and BMI has been reported in women with BMI < 30 kg/m^2^, no systematic study has been carried out in obese women [12]. The present study aimed to correct this deficiency and to assess other nutritional factors that may differ between obese and normal-weight mothers, notably human milk composition and volume.

Since leptin down-regulates appetite and increases energy expenditure, it may be expected to have a concentration-related impact on infant growth [13]. In a rat model, orally administered leptin has been shown to be absorbed by the immature stomach and may have a regulatory effect on weight gain in the offspring [14,15]. As maturation of the gastrointestinal tract proceeds, leptin absorption diminishes as stomach leptin secretion rises [14,15]. In human infants, plasma leptin concentration falls from birth to 4 weeks and rises afterwards with increasing weight [16]. The association between milk leptin concentration and IWG had been studied, but data are lacking on the association between leptin and exclusively breastfed IWG at 1 month in relation to the BMI status of the mother [17].

In the light of this, a study was designed with the main objective of comparing human milk leptin, macronutrient concentration, and volume in obese and normal-weight mothers whose infants were exclusively breastfed. The study population was controlled for age, ethnicity, pregnancy status and maternal educational level. As secondary aims, mother and infant characteristics were monitored within the study and related to physiological parameters and to milk composition.

## Materials and Methods

### Subjects

Mother-infant dyads were recruited between 22^nd^ February 2010 and 10^th^ September 2012, during postpartum hospitalization, in the maternity wards of the university hospital of Poitiers and the general hospital of Chatellerault, both in Western France. None of the maternity wards have the Baby-Friendly Hospital Initiative designation.

Obese mothers (BMI ≥ 30 kg.m^-2^) were matched with normal-weight mothers (18.5 ≤ BMI < 25 kg.m^-2^) for age (± 5 years), pregnancy status (parity, i.e., first child vs. second or more), ethnic origin (European descent, African descent, Asian descent), and maternal educational level (elementary or middle school, high school and higher). Mother/infant dyads with pre-existing chronic or gestational diseases, smokers during pregnancy, twin pregnancies, infants born preterm (*i*.*e*. with a gestational age < 37 weeks of amenorrhea), small for gestational age (birth weight < third centile for gestational age according to French references), or hospitalized in the neonatal period were excluded [18]. In utero growth restricted infants (with birth weight > third centile) or large for gestational age infants were not excluded. In a data subset of 1452 women who gave birth in both maternity wards, pre-gestational BMI ≤18.5 kg.m^-2^, 18.5≤ BMI <25 kg.m^-2^, 25≤ BMI <30 kg.m^-2^ and ≤30 kg.m^-2^ were present in respectively 10% (n = 144), 57% (n = 814), 22% (n = 317) and 11% (n = 160) of cases. Gestational or pre-gestational diseases were present in 111 women, more frequently in obese women (14 vs. 7%, p<0.01).

### Data Collection

**Fig 1:**
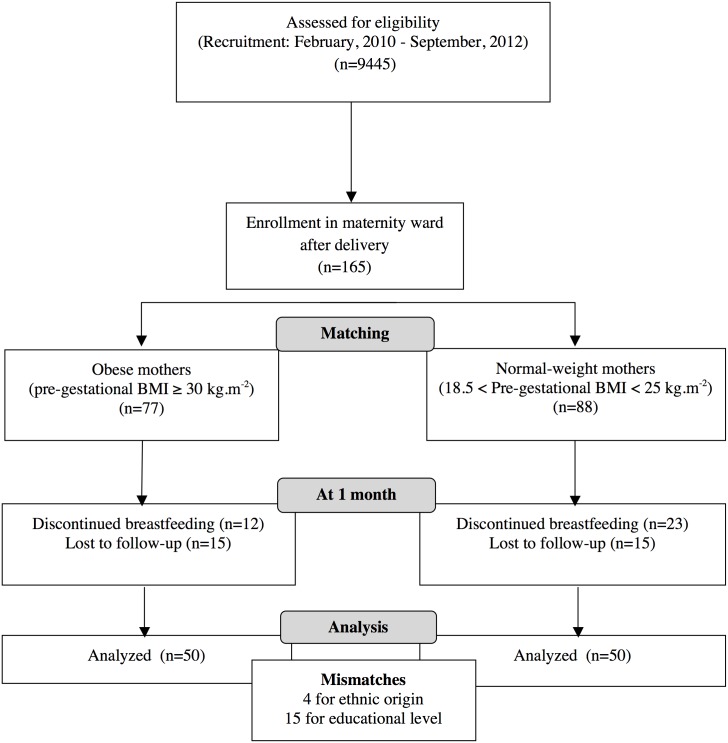
Flow diagram.

Only mother/infant dyads in which the infant remained exclusively breastfed throughout the first month postpartum were included in the study. Among the 165 women who gave consent, 2 groups of 50 mother-infant dyads (82 from Poitiers and 18 from Chatellerault) completed the entire study. Participating mothers and their infants were investigated at the clinical investigation center Inserm 1402 at 1 month postpartum. Thirty-five of 165 discontinued full breastfeeding and switched to non-exclusive breastfeeding or formula feeding and 30 left the study for other reasons (Fig 1). In each group, mothers were primiparous (n = 20) and multiparous (n = 30) with no mismatch; of European origin (n = 42) or African origin (n = 4) and 4 mismatches (European vs. African origin); middle school (n = 5) or higher (n = 30) and 15 mismatches (College vs. higher education level).

The hospital visit at which samples were taken was specific to this study and was always scheduled between 9 and 11 in the morning, in order to limit the effect of circadian variation in milk composition. Human milk was collected during the mother’s usual nursing time using a breast pump (Symphony, Medela^™^, Switzerland) from the breast opposite to the breast suckled by the infant. Milk collection lasted for as long as the infant suckled. Collected milk was blind-coded with a same identification number for each mother-infant dyad, aliquoted, and frozen at -80°C until analysis. A physical examination was performed on both mother and infant. Maternal height was measured with a stadiometer (Model HR 001, Tanita^™^, Leicester, England) and maternal weight was measured using electronic scales (Model 803, Seca^™^, Hamburg, Germany). Weight and height before pregnancy, and at delivery (measured during the 3 to 5 days of postpartum hospitalization) were obtained from medical files. Infant weight was measured using electronic Seca scales (Model 335, Seca^™^, Hamburg, Germany) and length using a steel somatometer (Model 207, Seca^™^, Hamburg, Germany). Weight gain from birth to 1 month was expressed in grams and in grams per day (weight gain velocity).

### Leptin Analysis

Leptin analysis can potentially be affected by different sample handling protocols [13]. The procedure adopted was based on that previously recommended [13]. All analyses were carried out on samples that were conserved frozen and only thawed just before analysis. The whole milk samples were vigorously vortexed to ensure sample homogeneity, in particular to disperse fat globules and were diluted 1:1 with assay buffer, as recommended in the assay instructions and in [13]. Leptin content was measured using Human leptin radioimmunoassay kit (Linco research Inc., Saint-Charles, MO, USA). All tubes were counted in a gamma counter and leptin concentrations were calculated using automated data reduction procedures. Analysis was performed in duplicate with repeatability below 10% and re-analyzed if above that cutoff. The detection limit was 0.5 ng.mL^-1^ with 100 μL samples and the limit of linearity was 100 ng.mL^-1^.

### Miris Analysis

Human milk was analyzed using a MIRIS^®^ human milk analyzer (MIRIS HMA) (MIRIS, Uppsala, Sweden), with a validated protocol [19]. Two samples each of 2 mL were thawed, warmed at 40°C and homogenized for 90 s using a Bioruptor^®^ sonicator (Diagenode, Liège, Belgium). Protein, lipid, carbohydrate, and dry matter content were determined and analyses were performed in duplicate. Milk energy content (kJ.100 mL^-1^) was calculated, based on energy values of 38.7, 16.7, and 18.4 kJ.g^-1^ for fat, carbohydrate and protein, respectively. The absence of drift in the MIRIS HMA measurement over time was checked using aliquots of frozen milk samples specifically prepared and stored for this purpose.

### Ethics

This study was conducted following the Declaration of Helsinki human subjects research guidelines. Ethics committee CPP Ouest III approved all procedures involving human subjects (September 7^th^, 2009). After explaining the study protocol to the mother, written consent was obtained from all participating mothers. This clinical trial was registered at the EudraCTClinical Trials Registry (eudract.ema.europa.eu) as 2009-A00912-55.

### Statistical Analysis

The number of mother-infant dyads to include was calculated to attain the primary aim of the study i.e. to demonstrate a higher human milk leptin concentration in the obese group. The inclusion of 50 obese and 50 normal-weight mothers allowed the detection of a difference of 0.15 ± 0.2 ng.mL^-1^ in milk leptin concentration (95% power, 5% significance level). Data are expressed as means ± standard deviation. Distributions were tested for normality using Shapiro-Wilk test. Comparisons between groups were performed using an unpaired t-test in the case of normality and a Wilcoxon rank sum test for parameters showing a non-normal distribution. Correlation analyses were performed on the whole dataset using Pearson’s coefficient. *P* values <0.05 were considered statistically significant. Results were adjusted for ethnicity and maternal educational level. All statistical analyses were computed using JMP 10.0.2 software (SAS Institute Inc., Cary, NC, USA).

## Results

### Characteristics of the Mothers

Compared with normal-weight mothers, obese mothers did not differ in age but were on average heavier, 2.5 cm shorter, and had a higher BMI (Table 1).

**Table 1:** Characteristics of the mothers (N = 100).

	Normal-weight mothers (18.5≤BMI<25 kg.m^-2^) (n = 50)	CI 95%	Obese mothers (BMI≥30 kg.m^-2^) (n = 50)	CI 95%	*p*
Age (years)*	30.6	29.3–31.8	30.8	29.4–32.2	0.59
**Pre-gestational weight (kg)**	59.3	57.9–60.7	90.8	87.7–94.0	<0.001
**Pre-gestational height (cm)**	165.7	164.0–167.4	163.2	161.6–164.8	0.01
**Pre-gestational BMI (kg.m**^**-2**^**)**	21.6	21.2–22.0	34.1	33.0–35.2	<0.001
**Maternal BMI at birth (kg.m**^**-2**^**)**	26.8	26.1–27.4	37.7 (49)	36.8–38.7	<0.001
**Maternal BMI at 1 month (kg.m**^**-2**^**)**	24.0	23.1–25.0	35.3	34.4–36.2	<0.001

Results are means and Confidence Interval 95%. The number of patients included in the mean is 50, unless indicated otherwise in brackets. The *p* is the t-test between pairs of obese/normal-weight mothers adjusted for ethnicity and educational level. BMI, Body mass Index (kg.m^-2^); CI 95%, Confidence Interval 95%;

*, non-normal distribution.

### Milk Composition and Volume

**Fig 2:**
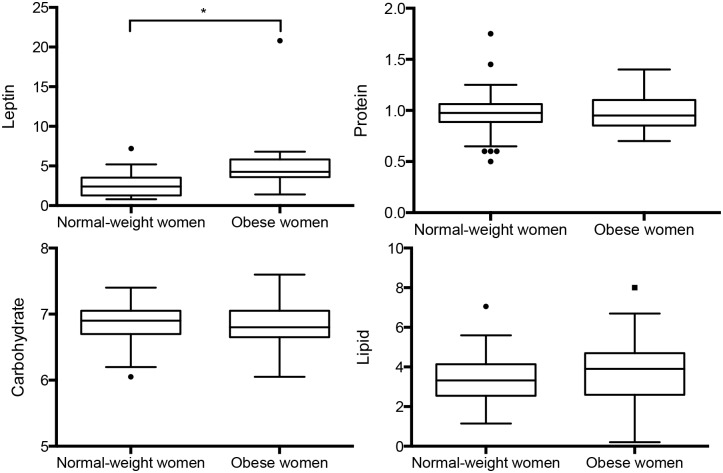
Comparison of human milk composition between normal-weight and obese women. Leptin in ng.mL^-1^, *p<0.001; Carbohydrates, Protein, Lipids in g.100mL^-1^, non significant.

**Fig 3:**
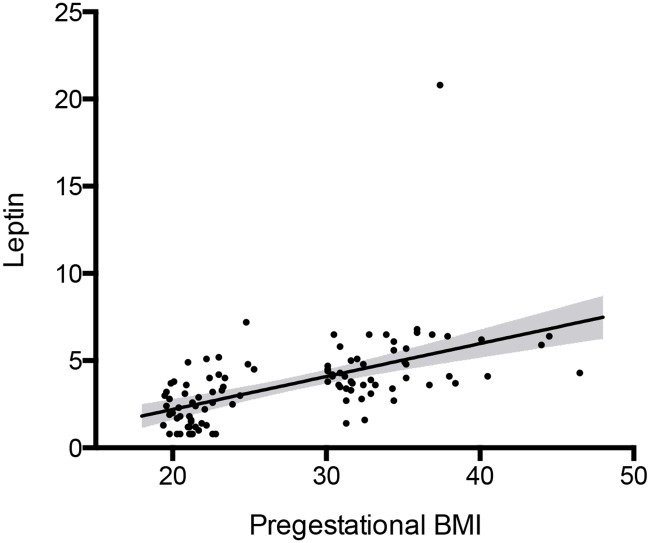
Correlation between milk leptin concentration and pre-gestational BMI. Leptin in ng.mL^-1^, BMI in kg.m^-2^; *r*^*2*^ = 0.33, *p*<0.001; line: linear regression line, grey area: confidence interval 95%.

Leptin concentration was significantly higher in milk from obese mothers (Table 2, Fig 2). The difference remained significant after removing the outlier in the obese group. No difference was found between groups for milk concentrations of protein, lipid or carbohydrate (Table 2, Fig 2) or for the calculated milk energy content. The volume and dry matter of milk collected at 1 breastfeeding were identical in the 2 groups. Milk leptin concentration was correlated to pre-gestational BMI (*r*^*2*^ = 0.33) (Fig 3).

**Table 2:** Milk composition and volume (N = 100).

	Normal-weight mothers (18.5≤BMI<25 kg.m^-2^) (n = 50)	CI 95%	Obese mothers (BMI≥30 kg.m^-2^) (n = 50)	CI 95%	*p*
**Leptin (ng.mL**^**-1**^**)** *	2.5	2.1–3.0	4.8	4.1–5.6	<0.001
**Protein (g.100mL**^**-1**^**)** *	1.0	0.9–1.0	1.0 (47)	0.9–1.0	0.94
**Lipid (g.100mL**^**-1**^**)**	3.4	3.0–3.7	3.7 (47)	3.2–4.2	0.55
**Carbohydrate (g.100mL**^**-1**^**)**	6.8	6.7–6.9	6.8 (47)	6.8–6.9	0.12
**Energy (kJ.100mL**^**-1**^**) [kcal.100mL**^**-1**^**]**	259 [61.9]	243–274 [58.1–65.6]	275 [65.7] (47)	259–291 [61.9–69.5]	0.33
**Dry matter (g.100mL**^**-1**^**)**	11.7	11.4–12.1	12.4 (47)	11.8–12.9	0.10
**Volume of milk (mL)**	53	48–58	47	43–51	0.27

Results are means and Confidence Interval 95%. The number of patients included in the mean is 50, unless indicated otherwise in brackets. The *p* is t-test between pairs of obese/normal-weight mothers adjusted for ethnicity and educational level. BMI, Body mass Index (kg.m-^2^); CI 95%, Confidence Interval 95%;

*, non-normal distribution.

### Characteristics of the Infants

The characteristics of the infants are presented in Table 3. We did not observe any difference between groups at birth and at 1 month regarding gestational age, gender, weight, length, head circumference, weight gain, weight gain velocity, or age.

**Table 3:** Characteristics of the infants (N = 100).

	Normal-weight mothers (18.5≤BMI<25 kg.m^-2^) (n = 50)	CI 95%	Obese mothers (BMI≥30 kg.m^-2^) (n = 50)	CI 95%	*p*
**At birth**					
Gestational age (weeks)*	40.1	39.7–40.4	40.0	39.7–40.4	0.53
Weight (g)*	3420	3288–3531	3462	3331–3594	0.84
**Length (cm)**	50.0	49.5–50.6	49.8	49.3–50.4	0.61
Head circumference (cm)*	34.8 (47)	34.4–35.3	34.6	34.2–35.0	0.85
**Male/Female**	24/26		30/20		0.37
**At 1 month**					
**Weight (g)**	4258	4098–4418	4212	4016–4408	0.67
**Length (cm)**	53.6 (49)	52.9–54.2	53.5	52.9–54.1	0.92
Head circumference (cm)*	37.0 (47)	36.6–37.4	36.7	36.3–37.1	0.40
**Weight gain (g)**	838	739–938	750	615–884	0.22
**Weight gain velocity (g.d**^**-1**^**)**	26.7	23.9–29.6	24.0	20.2–27.9	0.17

Results are means and Confidence Interval 95%. The number of patients included in the mean is 50, unless indicated otherwise in brackets. The *p* is t-test between pairs of obese/normal-weight mothers adjusted for ethnicity and educational level. BMI, Body mass Index (kg.m-^2^); CI 95%, Confidence Interval 95%;

*, non-normal distribution.

Infant mean age was 31.2 days [30.2;32.3] in the normal-weight group and 30.8 days [29.6;32.0] in the obese group, p = 0.59.

## Discussion

Milk leptin concentration was higher in obese than in normal-weight mothers and was positively correlated to pre-gestational BMI. We did not observe any difference in protein, lipid or carbohydrate content between the 2 groups.

Milk leptin concentration was nearly 2-fold higher in the milk of obese mothers compared with that of normal-weight mothers, confirming our working hypothesis. In a relatively recent review Andreas et al. (2014) drew together the data from 15 studies of breast milk leptin concentration and maternal BMI [12]. While 10 studies reported a correlation over the entire time range analyzed, 4 further found no correlation, while the others found a correlation shortly postpartum but none thereafter. At the age of 4 to 6 weeks, six studies found a correlation between maternal BMI and breast milk leptin concentration, four others found no relationship or only early correlation. The authors noted that there was a high degree of variability between the way that the studies were conducted with regard to sample size, collection, preparation and analysis of breast milk samples. Furthermore, the studies were only performed with normal-weight, overweight and slightly obese women. In these studies, most women were overweight but not obese and none included a specific group of obese women. Thus, the present study is the first to consider this group of women, and to show that for obese women the correlation between leptin concentration and BMI is very marked.

The strengths of the present study, in contrast to the prior studies, are that (i) it is specifically designed to compare obese (as against overweight) and normal-weight women, and (ii) it takes into account parameters such as age, pregnancy status, ethnic origin and maternal educational level. It lacks, however, details of food records and physical activity level. Recruitment was more difficult than expected and a consequence of this was that efficient matching was only possible for age and parity. For these reasons, mean values were tested using the unpaired t-test procedure adjusted for ethnicity and educational level. Some data had non normal distribution but this did not change the significance of the results. The leptin concentration was measured as a single assay point in this cross-sectional design. Measures repeated over time would have been interesting and added strength to the data set. However, we chose the endpoint of 1 month because breastfeeding prevalence in France falls after this period and it was the endpoint of our previous (less controlled) study where we found a difference in weight gain at 1 month [2,8]. We recognize that this single time-point sampling approach may add to variability, notably in preparation and homogenization. We therefore used a validated method to reduce this variability and 2 measures of the same sample with <10% inter-variability were conducted. In order to control for diurnal changes in breast milk composition, the milk collection was sampled in the morning, during the mother’s usual nursing time. Only one outlier in milk leptin concentration could be seen in the boxplot of the obese group but the significance remained unchanged when this point is removed.

The present study did not support the secondary hypothesis: that there is an association between human milk leptin concentration and IWG during the first month. This parameter had been previously included in a less tightly controlled investigation into obesity and breastfeeding [8]. However, in the present study the difference measured is less than 100 g, approximately half that observed in our previous study, and is not significant. A number of factors may have contributed to the discrepancy between the 2 studies. In the present study, mothers were recruited before initiating breastfeeding, and then involvement of participating mothers might have been stronger in the present study, with a possible impact on breastfeeding practices, particularly in obese women. Furthermore, power calculations were made for milk leptin concentration, the main outcome and underpowered secondary aims. Initiation of breastfeeding was similar in the 2 locations and in accordance with the mean percentage of breastfeeding initiation in France [20]. We did not observe any difference in the volume of milk collected on 1 breastfeeding and on the number of breastfeeding per day, 2 parameters that are approximate indices of daily human milk production. However, they require cautious interpretation, particularly when single-sample data is collected. We cannot exclude different milk production or quality between groups, although we did not observe any difference in protein, lipid or carbohydrate content between the 2 groups. Nevertheless, these parameters must be taken into account when assessing the effect of leptin on weight gain. In any case, from the previous study [8] and the present data, whether or not human milk leptin concentration has an effect on weight gain at 1 month is clearly marginal and a larger group of mother-infant dyads is necessary to answer definitively this question. Other adipocytokines such as adiponectin, resistin, TNF-α and IL-6 or appetite regulating peptides GLP-1, PYY and ghrelin were not measured in the present study, which did not involve any blood sampling, and their impact needs to be determined in parallel to that of leptin.

Over the period 1997 to 2006, the French triennial large-scale obesity study (OBEPI) reported an increasing prevalence of overweight and obesity in French women of child-bearing age, 20–39 years [2]. The proportion of women overweight increased from 11.1 to 13.6% and from 15.0 to 20.2% in the categories 20–29 years and 30–39 years, respectively. More worrying, however, was the doubling of obesity in both age categories: from 3.6 to 7.7% and from 6.5 to 13.7%, respectively, over this 10-year period [2]. Obesity therefore is an escalating public health concern in women, particularly since pre-gestational BMI is positively linked to the offspring’s adiposity and cardio-metabolic parameters at 32 y independently of gestational weight gain [21]. Understanding the impact of this on infant nutrition is therefore of pressing importance.

## Conclusion

The key finding of this study is that human milk leptin concentration at 1 month postpartum is significantly higher in obese than in normal-weight mothers. Furthermore, we did not observe any difference in macronutrient content of human milk between the 2 study groups. Although we have previously reported an indication that obesity and IWG may be linked, no association with differential weight gain in infants exclusively breastfed for 1 month was found in this more rigorous study but this needs to be revisited. Of greater importance, it remains to be established whether the higher leptin content impacts on infant growth beyond the 1-month of the study period.
